# Editorial: Autoimmune complications of modern cancer therapies

**DOI:** 10.3389/fimmu.2023.1357825

**Published:** 2024-01-05

**Authors:** Nora Möhn, Mirjam Renovanz, David Hagin, Thomas Skripuletz

**Affiliations:** ^1^ Department of Neurology, Hannover Medical School, Hannover, Germany; ^2^ Immune Cooperative Oncology Group, Comprehensive Cancer Center Hannover (ICOG-CCCH), Hannover, Germany; ^3^ Department of Neurology & Interdisciplinary Neuro-Oncology, University Hospital Tuebingen, Hertie Institute for Clinical Brain Research, Tuebingen, Germany; ^4^ Department of Neurosurgery, University Hospital Tuebingen, Tuebingen, Germany; ^5^ Department of Immunology, Tel Aviv Sourasky Medical Center, Tel Aviv, Israel

**Keywords:** irAE, biomarkers, immune checkpoint inhibitor (ICI), immunotherapy, JAK1 and JAK2 inhibitors

Modern immune therapies and especially immune-checkpoint inhibitors (ICI) have revolutionized the treatment of different tumor entities within the last ten years (1).

Since the approval of the first ICI, Ipilimumab, in 2011, the indications are steadily increasing, and the prognosis of several previously lethal tumor entities has been improved (2). ICI are monoclonal antibodies, that inhibit particular immune-checkpoint receptors like cytotoxic T-lymphocyte-associated protein 4 (CTLA-4), programmed cell death protein 1 (PD-1) or its ligand (PD-L1) leading to enhanced cytotoxic T cell activity, reversal of T-cell exhaustion and thus an augmented antitumor response (3). Consecutively, immune evasion of cancer cell populations can be prevented (4). However, this kind of nonspecific immune activation could also be directed against endogenous tissue and consequently trigger so called immune-related adverse events (irAE). IrAE are potentially serious autoimmune side effects that can affect almost every organ (5, 6). Regardless of which organ is affected, rapid and effective initiation of immunosuppressive therapy is critical in higher grade irAE. Corticosteroids are used as standard therapy to suppress the inflammatory reaction (6, 7). Concurrently, ICI therapy is usually either paused or stopped completely with a potentially negative impact on tumor outcome (4). With the increasing indications of ICI-therapy, the number of irAE is rising as well leading to the development of guidelines (8). An intensive monitoring of patients under ICI-therapy is necessary to detect and treat irAE as early as possible. Nevertheless, a solely clinical monitoring is often not sufficient to detect serious side effects in a timely manner or, in the best case, to prevent their occurrence (9).

In order to better understand and predict irAE, a precise clinical characterization is of great importance. The case reports published in this topic can be particularly helpful in this regard. At the same time, clinical colleagues can be guided by the therapeutic approach in the cases presented. The most common irAE are gastrointestinal or dermatological, so the published case reports are also representative in this respect. Fang et al. described a rare case of ICI-induced pancreatitis with type 1 diabetes mellitus, a combination that has only been reported three times before in the literature (10–12). An important finding of the authors is that male gender and the use of PD-1 antibodies may be risk factors for the development of ICI-induced pancreatitis and that, compared to other irAE, the use of steroids should be more restrictive in concomitant type 1 diabetes. Hepatitis is much more common than immunotherapy-induced pancreatitis. In this condition, therapy-refractory cases are repeatedly described. Zarrabi et al. gave an example of this in their case report. Following prolonged unsuccessful treatment with various immunosuppressants the described patient was treated with the anti-CD25 monoclonal antibody basiliximab which resulted in sustained resolution of her hepatitis without significant side effects. Basiliximab could therefore also be used as an individualized treatment attempt for other steroid-refractory irAE. In addition to gastrointestinal irAE, dermatological adverse events are particularly frequent under ICI therapy (13). However, there are also some impressive and rare examples among these. Nakamura et al. presented the case of a 73-year-old patient with metastatic esophageal squamous cell carcinoma who developed pemphigus vulgaris during combination therapy with ipilimumab and nivolumab. After cessation of ipilimumab and continuation of nivolumab monotherapy, the findings regressed under topical steroids, leading the authors to speculate that the CTLA-4 antibody may have been the trigger for the blistering disease in this case. In principle, early detection and treatment often improves the outcome in every case of immunotherapy-associated side effects. To achieve this, clinical and laboratory biomarkers as well as patient-specific markers are required. A Chinese research group under Liu et al. was able to show that the HLA profile of patients with ICI-induced diabetes mellitus differs from that of patients with non-therapy-associated type 1 diabetes, while the clinical features are rather similar. The fact that patients with ICI-associated diabetes mellitus exhibit low frequencies of type 1 diabetes susceptibility and high frequencies of protective HLA haplotypes indicates that this specific irAE represents a new model distinct from classical type 1 diabetes. Neurological adverse events (nAE) like encephalitis or peripheral neuropathy are of special interest, as they are relatively rare but often associated with increased morbidity and also mortality (14). In addition, they are rather difficult to diagnose and are therefore often detected relatively late (15). The search for new serum biomarkers is therefore of great importance. In their original work, Leonie Müller-Jensen et al. were able to show that the presence of neuromuscular autoantibodies has a high sensitivity and specificity for the diagnosis of ICI-induced myositis, myocarditis or myasthenia, and may also predict these. In a comprehensive review article within this research area, Williams et al. summarized the most important findings on tissue alterations in patients with irAE. An increased infiltration of CXCR3+ effector T cells in the tissue was identified as a common feature of dermatological, gastrointestinal and musculoskeletal irAE. However, the authors also note that the mechanisms underlying the individualized irAE-related tissue changes from person to person are still largely unknown and that genetic variants, the host microbiome, pre-existing immune disorders and stromal factors could play a role.

In the last article in the topic, Chen et al. provide a detailed description of the pharmacokinetic properties of golidocitinib, a selective JAK1 inhibitor. The results of the presented phase I studies “JACKPOT2 and JACKPOT3” indicate that there are no clinically relevant inter-ethnic differences with regard to the pharmacokinetics of the substance. Diet also had no significant influence. Importantly, there were no major side effects of golidocitinib in either study.

In summary, it is evident that the use of oncological immunotherapies and, in particular, their side effects are a highly topical issue. In addition to educational individual case reports, new potential biomarkers were introduced. These mark an important step towards patient-specific therapy and risk stratification and can serve as a starting point for further promising work.

## Author contributions

NM: Conceptualization, Writing – original draft. MR: Writing – review & editing. DH: Writing – review & editing. TS: Supervision, Writing – review & editing.
